# Developmental trajectories of visual acuity and associated determinants in Chinese primary school students: a longitudinal cohort study

**DOI:** 10.3389/fpubh.2026.1793830

**Published:** 2026-05-12

**Authors:** Zewei Chen, Fan Yang, Lin Yue, Dandi Chen, Mengrong Zhu

**Affiliations:** Department of Health Policy and Management, West China School of Public Health and West China Fourth Hospital, Sichuan University, Chengdu, China

**Keywords:** Chinese primary school students, LCGM, myopia, trajectory, visual acuity

## Abstract

**Objective:**

This study aims to explore different visual development trajectories among Chinese elementary school students and their risk factors.

**Methods:**

A longitudinal cohort study was conducted from 2024 to 2025, involving students from four grades (2020–2023) at a primary school in Chengdu, China. Participants underwent 10 waves of visual acuity examinations over the one-year period. Latent Class Growth Modeling (LCGM) was employed to classify visual acuity development trajectories. A comprehensive survey was administered to assess potential risk factors, and multinomial logistic regression was utilized to analyze the determinants of trajectory.

**Result:**

Three distinct trajectories were identified: “moderate-initial rapid decline” (Class 1, *n* = 683), “high-initial stable” (Class 2, *n* = 267), and “low-initial slow decline” (Class 3, *n* = 184). Higher grade levels and maternal myopia were significant predictors of the low-initial slow decline trajectory. Reading posture, bedtime, and frequency of health education sessions were significant factors influencing visual development trajectories.

**Conclusion:**

These findings highlight the importance of targeted, trajectory-based screening strategies in school settings. Early identification of high-risk subgroups—particularly those in higher grades with maternal myopia—may inform the development of precision interventions to mitigate myopia progression among Chinese primary school children.

## Introduction

The global burden and the current situation of myopia in China have become serious public health concerns, with prevalence rising sharply over the past few decades. It is projected that by 2050, nearly half of the world’s population will be affected by myopia, with a substantial increase in the proportion of high myopia (1). This phenomenon is particularly pronounced in East Asia. As a country with a high prevalence of myopia, China continues to face persistently high rates among children and adolescents, with trends toward earlier onset and greater severity (2).

Primary school age is a critical period for visual development and also a stage when myopia commonly emerges. Myopia leads to visual deterioration, which can affect children’s academic performance and quality of life. Severe high myopia may result in blinding complications such as retinal detachment and macular degeneration (3). In addition, previous studies have reported significant associations between severe refractive errors and increased risks of depression and anxiety disorders (4). Therefore, investigating the developmental patterns of visual acuity and their key influencing factors is essential for maintaining visual health among school-aged children.

Research on the factors influencing visual development has become extensive, covering multiple dimensions such as genetics, outdoor activities, near-work load, and environmental lighting. However, many studies describe visual development based on average trends observed in the overall sample (5–8). In reality, substantial heterogeneity exists in children’s visual development: at the same age, some children maintain stable vision, some experience gradual decline, while others exhibit more rapid deterioration. Analyses focusing solely on average trends may overlook such underlying heterogeneity, which can limit the identification of distinct developmental patterns and hinder a more nuanced understanding of their determinants.

To better characterize this heterogeneity, analytical approaches that can capture differences in developmental trajectories are needed. In this study, Latent Class Growth Modeling (LCGM) was employed to identify subgroups of children with distinct visual acuity trajectories over time, rather than assuming a single uniform pattern of change. This approach allows for a more detailed description of variation in visual development and facilitates the identification of subgroups that may be at higher risk of adverse visual outcomes. Although LCGM has been widely applied in psychological research (9, 10), its application in studies of visual acuity development remains relatively limited, particularly in relation to examining factors associated with different trajectory patterns.

Based on a one-year longitudinal survey of 1,256 Chinese primary students, this study aims to support precise prevention by identified distinct visual development progression pathways.

The study objectives are: (1) to employ Latent Class Growth Modeling (LCGM) to identify and characterize latent subgroups of visual acuity development trajectories among primary school students; and (2) building upon this trajectory classification, to examine the extent to which personal (age, gender), environmental/behavioral (screen time, lighting), and lifestyle factors (sleep, health education) differentially predict membership in each identified subgroup, with the aim of informing subgroup-specific prevention strategies.

## Methods

### Participants

This study was conducted at a primary school in Chengdu, Sichuan Province, China. The cohort included a total of 1,256 students across four grade levels (2020–2023), excluding the entry-level (first grade, 2024) and graduating (sixth grade, 2019) classes to minimize attrition. The longitudinal follow-up spanned from March 2024 to March 2025. During this period, participants underwent 10 waves of visual acuity examinations at intervals of 1 to 2 months. Additionally, risk factors associated with visual acuity development were assessed via an online survey platform (*Wenjuanxing*, https://www.wjx.cn/). This questionnaire, administered to parents in December 2024, comprised 21 items across 7 distinct categories. Ethical approval was obtained through Ethics Committee of West China Fourth Hospital and West China School of Public Health. Sichuan University (Gw112024179).

**Figure 1:**
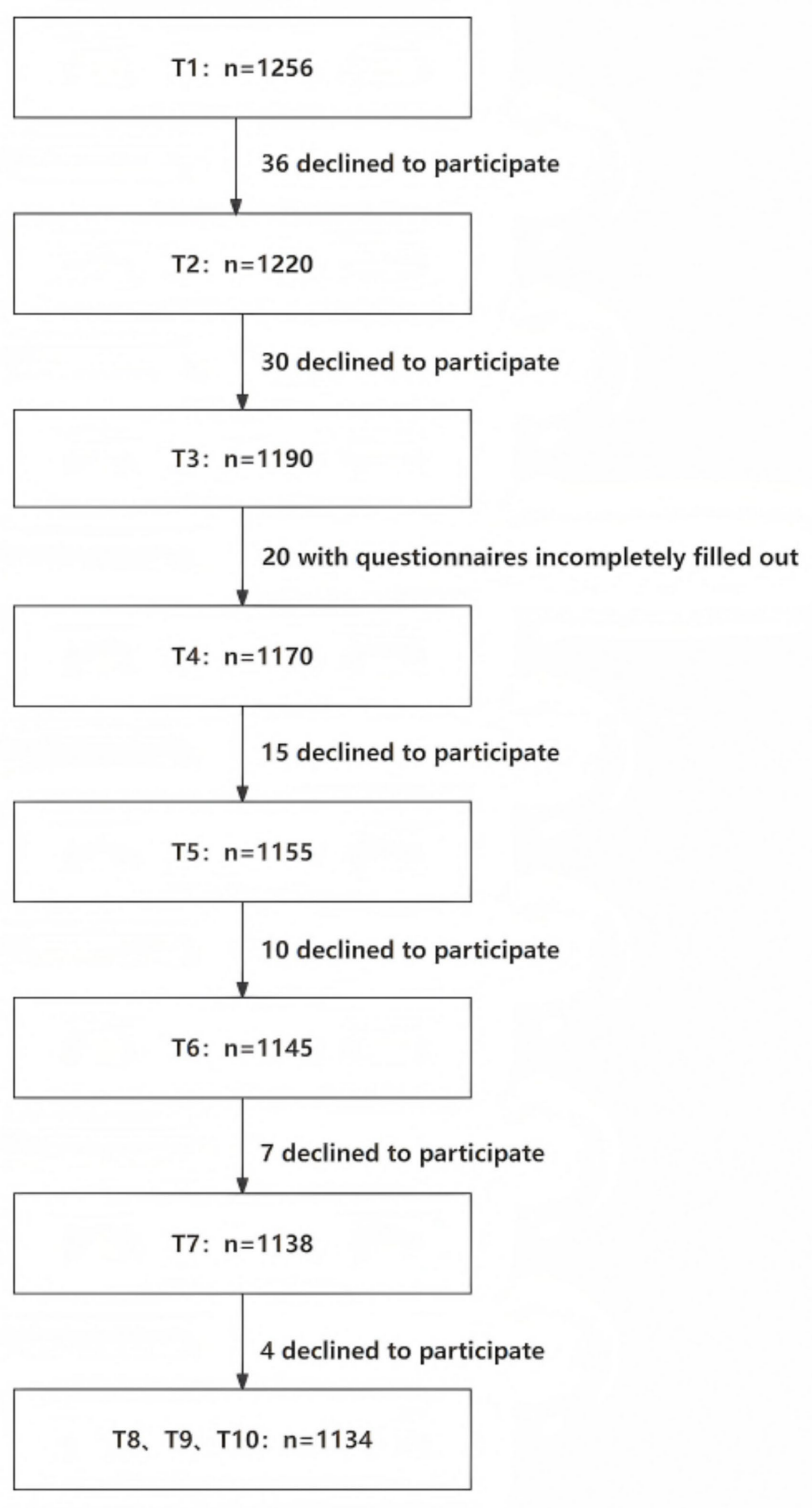
Flowchart of participant follow-up.

Both the visual acuity examination results and questionnaire datasets underwent systematic cleaning. The procedure involved excluding participants lost to follow-up, removing incomplete questionnaire responses and duplicate records, and verifying data logical consistency. Subsequently, the cleaned questionnaire data were merged with the visual acuity data using unique personal identifiers. These steps resulted in a final analytic dataset of 1,134 students. Each participant in this final cohort possessed valid, paired data for both visual metrics and questionnaire responses. The detailed data processing flow is illustrated in Figure 1.

### Measures

#### Visual acuity examinations

Visual acuity was assessed across all 10 waves using the Standard for Logarithmic Visual Acuity Chart (GB/T 11533-2011), issued by the National Health Commission of China (11). This Standard specifies the design criteria, printing specification, use methods, and visual acuity statistical methods, etc. of visual acuity charts. Under this standard, normal visual acuity is defined as the ability to resolve a visual angle of 1 min of arc. This is recorded as a score of 5.0 on the 5-point scale, which is equivalent to a decimal acuity of 1.0, or a fractional acuity of 6/6 or 20/20.

The participant’s visual level was expressed as mean visual acuity of both eyes, given there was no statistically significant difference between the left and right eye examination results (*p* = 0.670).

#### Survey

Questionnaire Survey included demographic and anthropometric characteristics, family history and ocular conditions, near-work behaviors, electronic device exposure, sleep and circadian behaviors, outdoor and dietary behaviors, as well as vision care and health education factors. As shown in Table 1.

**Table 1:** Questionnaire survey.

No.	Independent variable category	Independent variables included
1	Demographics and anthropometrics	Weight, height, grade, and sex
2	Genetic and ocular history	Parental myopia and other eye diseases
3	Near-work and reading habits	Daily study time, reading in a lying position, and near-work duration during holidays
4	Electronic device exposure	Daily screen time, use of electronic devices after lights-off, and increased screen use during holidays
5	Sleep and circadian behaviors	Nighttime sleep duration, bedtime, nap duration, and reduced sleep during holidays
6	Outdoor activity and dietary factors	Frequency of outdoor activities and anthocyanin-rich food intake
7	Preventive care and health education	Regular vision examinations and vision health education

### Data analysis

Latent Class Growth Modeling (LCGM) was employed to identify heterogeneous trajectories of visual acuity development. This model assumes the existence of *K* unobserved latent classes within the population, where individuals in the same class follow a similar growth pattern over time.

All analyses were performed using Mplus Version 8.3, utilizing Full Information Maximum Likelihood (FIML) estimation to handle longitudinal missing data. We fitted models ranging from 1 to 4 classes (*K* = 1 to *K* = 4). The optimal number of classes was determined based on a comprehensive evaluation of statistical indices and substantive interpretability:

*Bayesian Information Criterion (BIC)*: A lower BIC value indicates a better balance between model fit and parsimony;

*Entropy*: Measures classification accuracy, ranging from 0 to 1, with values above 0.70 typically indicating good classification;

*Average Posterior Probability (AvePP)*: The average probability of correctly classifying members within each category, which should exceed 0.80;

*Lo–Mendell–Rubin Likelihood Ratio Test (LMR-LRT)*: Tests whether the *K*-category model is significantly better than the *K* − 1 category model (*p* < 0.05);

*Bootstrap Likelihood Ratio Test (BLRT)*: Tests whether the *K*-class model fits significantly better than the *K* − 1-class model using a bootstrap procedure, with *p* < 0.05 supporting the more complex model.

Interpretability and Sample Size: Each class should be substantively meaningful and contain a reasonable proportion of the total sample (typically >5%).

To investigate baseline predictors associated with distinct visual acuity developmental trajectories, we extracted the final LCGM classification results from Mplus. To examine the factors associated with trajectory class membership, a multinomial logistic regression model was fitted, with trajectory class as the dependent variable. The model can be expressed as Equation 1. where *Y* represents trajectory class membership, *k* denotes each non-reference class, and $X_{1}$ to $X_{p}$ represent the independent variables included in the model, such as grade, maternal myopia, reading posture, sleep time, and other variates. Results were reported as Odds Ratios (OR) with 95% Confidence Intervals (CI). An independent variable was considered a significant predictor of a specific trajectory if its 95% CI did not include. *p* < 0.05 is considered statistically significant.


$$\log(\frac{P(Y=k)}{P(Y=\text{reference})})=\beta_{0}+\beta_{1}X_{1}+\beta_{2}X_{2}+\cdots +\beta_{p}X_{p}$$ (1)

Considering the multiple comparisons involved in the multinomial logistic regression, the Benjamini–Hochberg false discovery rate (FDR) method was applied to control for type I error, with a *q*-value <0.05 considered statistically significant.

Benjamini–Hochberg procedure:

Let p (1) ≤p (2) ≤⋯≤p (*m*) be the ordered *p*-values of m hypothesis tests. For a given false discovery rate level Q (e.g., Q=0.05 ), define:


$$k=\max\{i:p_{\left(i\right)}\leq \frac{i}{m}\cdot Q\}$$ (2)

Then reject all hypotheses H(1),…,H(k) . The adjusted p -value ( q -value) for each hypothesis is defined as:


$$q_{\left(i\right)}=\min(\min_{j\geq i}\{\frac{m}{j}\cdot p_{\left(j\right)}\},1)$$ (3)

A hypothesis is considered statistically significant if q(i)<Q .

## Results

### Questionnaire survey and vision acuity examination results

Table 2 summarizes the questionnaire responses and visual acuity test results for t1–t10 for the children, categorized by the three visual acuity trajectories. The descriptive statistics cover independent variables across 7 major domains and 21 sub-categories. We examined the distribution of key variables across the three trajectory classes using one-way ANOVA. The results showed that most variables were similarly distributed among the three groups, with the exception of grade, which demonstrated a statistically significant difference.

**Table 2:** Characteristics and longitudinal visual acuity variations among the three identified trajectory classes.

Factors	Sum	Class1	Class2	Class3	Test statistic	*p*-value
Demographics and anthropometrics						
Weight	32.20 ± 9.96	32.26 ± 10.02	32.35 ± 10.03	31.73 ± 9.64	0.25	0.778
Height	136.27 ± 9.46	136.1 ± 9.00	136.6 ± 10.20	136.5 ± 9.80	0.333	0.717
Grade					192.292	<0.001
2020	283	105	71	107		
2021	252	131	67	54		
2022	260	176	66	18		
2023	339	271	63	5		
Sex					1.01	0.604
Female	534	329	119	86		
Male	600	354	148	98		
Genetic and ocular history						
Mom myopia					4.854	0.088
No	563	352	133	78		
Yes	571	331	134	106		
Dad myopia					1.875	0.392
No	586	349	147	90		
Yes	548	334	120	94		
Other eye diseases					0.131	0.937
No	1,035	625	243	167		
Yes		58	24	17		
Near-work and posture						
Daily study time					2.041	0.36
<1 h	246	153	59	34		
1-2 h	643	379	148	116		
2-3 h	214	133	50	31		
>3 h	31	18	10	3		
Reading in a lying position					1.7	0.427
Hardly	482	278	116	88		
Occasionally	607	382	140	85		
Always	45	23	11	11		
Near-work duration during holidays					1.58	0.454
<30 min	253	159	57	37		
30-60 min	620	374	141	105		
1-2 h	192	110	53	29		
2-3 h	49	30	10	9		
>3 h	20	10	6	4		
Electronic device exposure						
Daily screen time					3.159	0.206
<0.5 h	815	499	181	135		
0.5-1 h	274	157	72	45		
>1 h	45	27	14	4		
Use of electronic devices after lights-off					3.514	0.173
No	903	532	222	149		
Yes	231	151	45	35		
Increased screen use during holidays					0.295	0.863
No	375	224	87	64		
Yes	759	459	180	120		
Sleep and circadian						
Night time sleep duration					0.204	0.903
≤8 h	218	134	49	35		
>8 h	916	549	218	149		
Bedtime					3.391	0.184
Before 21:00	63	38	13	12		
21:00–22:00	784	471	198	115		
After 22:00	287	174	56	57		
Nap duration					1.061	0.97
Hardly	761	459	178	124		
≤0.5 h	224	133	53	38		
>0.5 h	149	91	36	22		
Reduced sleep during holidays					1.835	0.399
No	857	507	209	141		
Yes	277	176	58	43		
Protective lifestyle factors						
Frequency of outdoor activities					1.42	0.492
Campus activities only	947	571	218	158		
Almost daily	187	112	49	26		
Anthocyanin-rich food intake					1.428	0.808
Hardly	675	404	163	108		
1-2 times per week	366	223	84	59		
3–5 times per week	66	39	14	13		
Almost daily	27	17	6	4		
Preventive care and education						
Regular vision examinations					2.064	0.356
No	881	528	215	138		
Yes	253	155	52	46		
Vision health education					2.856	0.24
Hardly	561	329	136	96		
Occasionally	458	272	112	74		
Always	115	82	19	14		
Visual acuity						
t1	4.995 ± 0.245	5.025 ± 0.151	5.197 ± 0.120	4.591 ± 0.196	1320.275	<0.001
t2	4.986 ± 0.257	5.009 ± 0.149	5.223 ± 0.095	4.552 ± 0.196	1963.296	<0.001
t3	4.986 ± 0.258	5.006 ± 0.140	5.236 ± 0.094	4.545 ± 0.198	2178.196	<0.001
t4	4.986 ± 0.256	5.002 ± 0.137	5.244 ± 0.076	4.549 ± 0.192	2585.664	<0.001
t5	4.955 ± 0.258	4.959 ± 0.123	5.243 ± 0.074	4.523 ± 0.200	2943.016	<0.001
t6	4.935 ± 0.264	4.928 ± 0.139	5.242 ± 0.072	4.512 ± 0.189	3052.559	<0.001
t7	4.901 ± 0.278	4.885 ± 0.169	5.223 ± 0.090	4.493 ± 0.201	2318.269	<0.001
t8	4.830 ± 0.304	4.790 ± 0.182	5.212 ± 0.119	4.427 ± 0.210	1713.085	<0.001
t9	4.789 ± 0.313	4.733 ± 0.189	5.191 ± 0.139	4.411 ± 0.235	1288.641	<0.001
t10	4.710 ± 0.335	4.612 ± 0.196	5.179 ± 0.158	4.394 ± 0.238	948.672	<0.001

### Trajectory of children’s visual development

As shown in Table 3. The optimal number of latent classes was determined through a comprehensive evaluation of fit indices, classification accuracy, and interpretability. We selected the 3-class solution as the best-fitting model. The 4-class model was rejected despite a lower BIC because it failed the LMR test (*p* = 0.3674) and contained a small subgroup (<5%). In contrast, the 3-class model showed a substantial improvement over the 2-class model (∆BIC > 3,600). Although the LMR was non-significant (*p* = 0.1338), we relied on the highly significant BLRT (*p* < 0.001), which is widely regarded as a superior metric to LMR-LRT for detecting the correct number of classes (12). This model also exhibited excellent classification precision (Entropy = 0.969) and adequate class sizes (>16%).

**Table 3:** Model fit indices for the latent class growth models with different numbers of classes.

*K*	BIC	Entropy	AvePP (min–max)	LMR-LRT	BLRT	*n*
1	4102.791	—	1	—	—	1,134
2	−6812.956	0.985	0.993–0.996	*p* = 0.0000^*^	*p* = 0.0000^*^	882,252
3	−10430.365	0.969	0.967–0.991	*p* = 0.1338	*p* = 0.0000^*^	683,267,184
4	−12700.789	0.974	0.977–0.988	*p* = 0.3674	*p* = 0.0000^*^	164,646,260,64

The trajectories of visual acuity differed markedly across the three classes over time. Class 2 consistently exhibited the highest level of visual acuity throughout all time points, remaining relatively stable with only a slight decline from 5.197 at baseline (t1) to 5.179 at t10. In contrast, Class 1 showed a moderate initial level of visual acuity (5.025 at t1) followed by a gradual and continuous decline over time, reaching 4.612 at t10. The downward trend became more pronounced after t6, indicating an accelerated deterioration in later stages. Class 3 had the lowest baseline visual acuity (4.591 at t1) and maintained a persistently low level throughout the follow-up period, with a mild but steady decrease to 4.394 at t10. Compared with the other classes, this group demonstrated a consistently disadvantaged visual trajectory.

**Figure 2:**
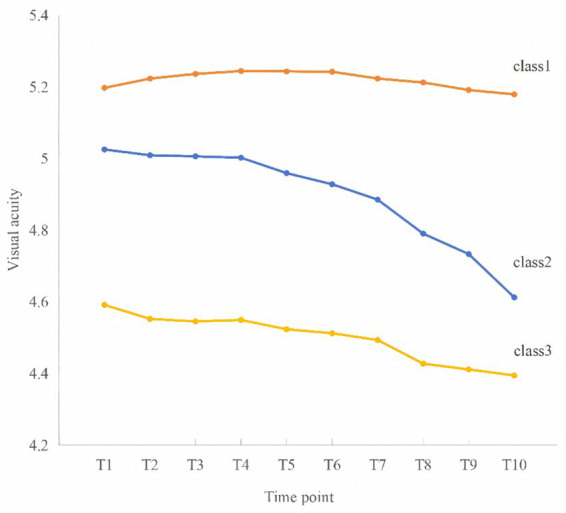
Visual acuity trajectories of class1–3.

Overall, these findings highlight distinct patterns of visual acuity development, trajectories were named based on their intercept (visual acuity at t1) and slope (trend over the follow-up duration). This classification yielded three distinct groups shown in Figure 2: “moderate-initial rapid decline” (Class 1, *n* = 683), “high-initial stable” (Class 2, *n* = 267), and “low-initial slow decline” (Class 3, *n* = 184).

### Association between myopia influencing factors and vision development trajectory

As shown in Table 4, after adjusting for confounding factors, the multinomial logistic regression analysis revealed several significant predictors of trajectory membership:

**Table 4:** Multinomial logistic regression analysis of factors associated with visual acuity trajectory classes.

Variables	*B* (SE)	CLASS1 vs. CLASS3 (ref) OR (95% CI)	*p*	*B* (SE)	CLASS2 vs. CLASS3 (ref) OR (95% CI)	*p*	*B* (SE)	CLASS1 vs. CLASS2 (ref) OR (95% CI)	*p*
Demographics and anthropometrics									
Weight	0.024 (0.013)	1.024 (0.998, 1.051)	0.072	0.018 (0.014)	1.019 (0.991, 1.047)	0.188	−0.005 (0.009)	0.995 (0.977, 1.013)	0.572
Height	−0.013 (0.013)	0.987 (0.962, 1.012)	0.297	−0.005 (0.014)	0.995 (0.968, 1.022)	0.715	0.008 (0.010)	1.008 (0.989, 1.028)	0.405
Grade (ref: 2023)									
2020	−4.407 (0.493)	0.012 (0.005, 0.032)	<0.001***	−3.262 (0.509)	0.038 (0.014, 0.104)	<0.001***	1.145 (0.217)	3.141 (2.051, 4.811)	<0.001***
2021	−3.375 (0.495)	0.034 (0.013, 0.090)	<0.001***	−2.495 (0.514)	0.082 (0.030, 0.226)	<0.001***	0.879 (0.214)	2.409 (1.584, 3.664)	<0.001***
2022	−1.869 (0.526)	0.154 (0.055, 0.432)	<0.001***	−1.366 (0.546)	0.255 (0.088, 0.744)	**0.012**	0.503 (0.208)	1.654 (1.101, 2.486)	**0.015**
Sex (ref: male)									
Female	0.209 (0.197)	1.232 (0.838, 1.813)	0.289	0.013 (0.213)	1.013 (0.667, 1.537)	0.953	−0.196 (0.155)	0.822 (0.606, 1.113)	0.205
Genetic and ocular history									
Maternal myopia (ref: yes)									
No	0.441 (0.206)	1.554 (1.039, 2.325)	**0.032**	0.271 (0.222)	1.312 (0.849, 2.027)	0.221	−0.170 (0.161)	0.844 (0.616, 1.156)	0.29
Paternal myopia (ref: yes)									
No	0.133 (0.205)	1.142 (0.764, 1.707)	0.517	0.278 (0.222)	1.321 (0.856, 2.039)	0.209	0.146 (0.161)	1.157 (0.844, 1.584)	0.364
Other eye diseases (ref: yes)									
No	0.124 (0.349)	1.132 (0.571, 2.243)	0.722	−0.039 (0.374)	0.962 (0.462, 2.004)	0.918	−0.163 (0.274)	0.850 (0.497, 1.454)	0.553
Near-work and posture									
Daily study time (ref: >3 h)									
<1 h	−0.787(0.775)	0.455 (0.100–2.078)	0.31	−1.2 (0.79)	0.301 (0.064–1.419)	0.129	−0.412 (0.465)	0.662 (0.266–1.648)	0.376
1-2 h	−1.048(0.749)	0.351 (0.081–1.520)	0.161	−1.469 (0.761)	0.230 (0.052–1.023)	0.054	−0.421 (0.444)	0.656 (0.275–1.568)	0.344
2-3 h	−0.39(0.764)	0.677 (0.151–3.029)	0.61	−0.866 (0.779)	0.421 (0.091–1.936)	0.266	−0.476 (0.459)	0.621 (0.252–1.528)	0.3
Reading in a lying position (ref: always)									
Hardly	1.438 (0.480)	4.210 (1.643, 10.792)	0.003**	0.985 (0.518)	2.678 (0.970, 7.394)	0.057	−0.453 (0.404)	0.636 (0.288, 1.405)	0.263
Occasionally	1.770 (0.471)	5.870 (2.332, 14.775)	<0.001***	1.170 (0.508)	3.222 (1.190, 8.721)	**0.021**	−0.600 (0.397)	0.549 (0.252, 1.195)	0.131
Near-work duration during holidays (ref: >3 h)									
<30 min	0.797 (0.713)	2.219 (0.549, 8.974)	0.263	0.521 (0.753)	1.685 (0.385, 7.371)	0.489	−0.276 (0.567)	0.759 (0.250, 2.305)	0.627
30–60 min	0.787 (0.692)	2.197 (0.566, 8.532)	0.255	0.502 (0.729)	1.651 (0.395, 6.897)	0.492	−0.285 (0.553)	0.752 (0.254, 2.221)	0.605
1-2 h	0.411 (0.714)	1.509 (0.372, 6.110)	0.565	0.498 (0.749)	1.645 (0.379, 7.144)	0.506	0.087 (0.567)	1.091 (0.359, 3.316)	0.879
2-3 h	0.663 (0.794)	1.940 (0.409, 9.196)	0.404	0.342 (0.856)	1.408 (0.263, 7.541)	0.689	−0.320 (0.661)	0.726 (0.199, 2.652)	0.628
Electronic device exposure									
Daily screen time (ref: >1 h)									
<0.5 h	−0.737 (0.619)	0.478 (0.142, 1.609)	0.234	−1.101 (0.637)	0.332 (0.095, 1.158)	0.084	−0.364 (0.374)	0.695 (0.334, 1.447)	0.33
0.5–1 h	−1.034 (0.636)	0.356 (0.102, 1.237)	0.104	−1.040 (0.654)	0.353 (0.098, 1.272)	0.111	−0.007 (0.387)	0.993 (0.465, 2.123)	0.986
Use of electronic devices after lights-off (ref: yes)									
No	0.028 (0.257)	1.028 (0.621, 1.703)	0.913	0.390 (0.286)	1.477 (0.843, 2.586)	0.173	0.362 (0.205)	1.436 (0.960, 2.147)	0.078
Increased screen use during holidays (ref: yes)									
No	−0.131 (0.212)	0.877 (0.579, 1.328)	0.535	−0.108 (0.229)	0.898 (0.573, 1.407)	0.638	0.024 (0.168)	1.024 (0.737, 1.423)	0.888
Sleep and circadian									
Night time sleep duration (ref: >8 h)									
≤8 h	−0.304 (0.262)	0.738 (0.442, 1.232)	0.245	−0.224 (0.284)	0.800 (0.459, 1.394)	0.43	0.080 (0.201)	1.084 (0.730, 1.608)	0.69
Bedtime (ref: after 22:00)									
Before 21:00	0.085 (0.446)	1.089 (0.455, 2.609)	0.848	0.298 (0.503)	1.347 (0.502, 3.612)	0.554	0.213 (0.385)	1.237 (0.581, 2.632)	0.581
21:00–22:00	0.259 (0.229)	1.295 (0.827, 2.027)	0.258	0.640 (0.255)	1.896 (1.150, 3.128)	**0.012**	0.381 (0.195)	1.464 (0.999, 2.147)	0.051
Nap duration (ref: >0.5 h)									
Hardly	−0.301 (0.295)	0.740 (0.415, 1.320)	0.308	−0.259 (0.320)	0.772 (0.412, 1.445)	0.418	0.041 (0.229)	1.042 (0.666, 1.632)	0.856
≤0.5 h	−0.467 (0.343)	0.627 (0.320, 1.229)	0.174	−0.312 (0.371)	0.732 (0.354, 1.515)	0.401	0.155 (0.268)	1.168 (0.690, 1.976)	0.563
Reduced sleep during holidays (ref: yes)									
No	0.038 (0.232)	1.039 (0.659, 1.637)	0.871	0.226 (0.256)	1.253 (0.759, 2.069)	0.377	0.188 (0.188)	1.207 (0.835, 1.743)	0.317
Protective lifestyle factors									
Frequency of outdoor activities (ref: almost daily)									
Campus activities only	−0.162 (0.272)	0.850 (0.499, 1.448)	0.55	−0.263 (0.289)	0.769 (0.437, 1.355)	0.363	−0.100 (0.203)	0.905 (0.608, 1.346)	0.621
Anthocyanin-rich food intake (ref: almost daily)									
Hardly	0.915 (0.653)	2.496 (0.694, 8.977)	0.161	0.831 (0.710)	2.296 (0.571, 9.228)	0.242	−0.084 (0.503)	0.920 (0.343, 2.464)	0.868
1-2 times per week	0.835 (0.661)	2.304 (0.630, 8.420)	0.207	0.673 (0.719)	1.960 (0.479, 8.025)	0.349	−0.162 (0.512)	0.851 (0.312, 2.320)	0.752
3–5 times per week	0.570 (0.750)	1.768 (0.407, 7.684)	0.447	0.501 (0.817)	1.650 (0.333, 8.185)	0.54	−0.069 (0.591)	0.933 (0.293, 2.973)	0.907
Preventive care and education									
Regular vision examinations (ref: yes)									
No	0.053 (0.252)	1.055 (0.644, 1.727)	0.832	0.279 (0.275)	1.322 (0.771, 2.265)	0.31	0.226 (0.201)	1.253 (0.846, 1.857)	0.261
Vision health education (ref: always)									
Hardly	−0.706 (0.362)	0.494 (0.243, 1.003)	0.051	−0.127 (0.411)	0.881 (0.393, 1.972)	0.757	0.579 (0.289)	1.784 (1.012, 3.146)	**0.046**
Occasionally	−0.692 (0.361)	0.500 (0.247, 1.015)	0.055	−0.112 (0.410)	0.894 (0.400, 1.998)	0.785	0.581 (0.288)	1.787 (1.017, 3.141)	**0.044**

Bold *p*-values indicate statistical significance in the unadjusted multivariable logistic regression. Significance levels were determined after Benjamini–Hochberg false discovery rate (FDR) correction for multiple comparisons (*Q* = 0.05). ***q < 0.001; **q < 0.01; *q < 0.05 (adjusted *p*-value).

*Grade Level*: Higher grade levels were associated with a significantly reduced likelihood of entering Class 1 and Class 2 compared to Class 3 (Grade 2020: OR = 0.012, *p* < 0.001; Grade 2021: OR = 0.034, *p* < 0.001; Grade 2022: OR = 0.154, *p* < 0.001). However, in comparison between Class 1 and Class 2, students in higher grades were more likely to belong to Class 1 (Grade 2020: OR = 3.143, *p* < 0.001; Grade 2021: OR = 2.409, *p* < 0.001; Grade 2022: OR = 1.654, *p* = 0.015). Consequently, the risk hierarchy for senior students followed the pattern: Class 3 > Class 1 > Class 2.

*Maternal Myopia*: Students whose mothers were not myopic had a higher risk of belonging to Class 1 than Class 3 (OR = 1.554, 95% CI: 1.039–2.325, *p* = 0.032).

*Reading Posture*: The frequency of reading while lying down significantly influenced trajectory assignment. Compared to Class 3, students who “hardly” lay down to read had 4.2 times the odds of being in Class 1 (OR = 4.210, 95% CI: 1.643–10.792, *p* = 0.003). Those who “occasionally” lay down showed elevated risks for both Class 1 (OR = 5.870, *p* < 0.001) and Class 2 (OR = 3.222, *p* = 0.021) relative to Class 3.

*Sleep Time*: A bedtime between 21:00 and 22:00 increased the likelihood of membership in Class 2 compared to Class 3 (OR = 1.896, 95% CI: 1.150–3.128, *p* = 0.012).

*Health Education*: The frequency of health education was a significant factor. Compared to Class 2, students who “hardly” or only “occasionally” received health education faced an increased risk of falling into Class 1, with odds increasing by 78.4% (OR = 1.784, *p* = 0.046) and 78.7% (OR = 1.787, *p* = 0.044), respectively.”

## Discussion

Rather than focusing solely on aggregate risk factors at the population level, this study applied Latent Class Growth Modeling (LCGM) to identify subgroups with distinct patterns of visual acuity development. Six key findings emerged: (1) The visual developmental trajectories of myopia among Chinese primary school students can be categorized into three distinct classes: “moderate-initial rapid decline”, “high-initial stable”, and “low-initial slow decline”. (2) Grade level acted as an independent factor, with higher grades more likely to be associated with the “low-initial slow decline” trajectory. (3) Maternal myopia was established as a determinant of the “low-initial slow decline” class. (4) Behavioral analysis revealed that a low frequency of supine reading (reading while lying down) was significantly linked to the “low-initial slow decline” pattern. (5) Optimal sleep timing (21:00–22:00) was associated with the favorable “high-initial stable” trajectory. (6) Conversely, limited health education was identified as a risk factor for the “moderate-initial rapid decline” progression.

The identification of three distinct visual acuity trajectory classes has important real-world implications. The “moderate initial–rapid decline” group may represent a high-risk population for accelerated visual deterioration, highlighting the need for early monitoring and timely intervention. In contrast, the “high-initial stable” group appears to maintain relatively good visual acuity over time, suggesting a lower priority for intensive intervention. The “low-initial slow decline” group, although relatively stable, consistently exhibits poorer visual acuity and may benefit from sustained attention and long-term management. This classification provides a more nuanced understanding of heterogeneity in visual development and may help inform targeted prevention strategies and resource allocation in child vision care.

These findings corroborate and expand upon prior literature regarding the onset and progression of myopia. Specifically, our observation that senior students are more likely to fall into the low-initial group aligns with the conclusions of Han et al. (13), who reported that myopia progression rates generally decelerate with age. Younger children (around 7 years old, comparable to the Grade 2023 cohort in our study) typically exhibit higher annual progression rates, whereas older children tend to show a plateauing effect.

This age-dependent pattern may be attributed to ocular maturation. As students age, the extensibility of the scleral wall decreases (increased scleral rigidity), which physically limits the rate of axial elongation and slows progression. Conversely, the low-initial characteristic reflects cumulative environmental influences. Senior primary students in China face intense academic pressure associated with the transition to junior middle school (“Xiao Sheng Chu”) (14). The resulting prolonged duration of near-work activities contributes to poorer baseline visual acuity. Therefore, the interplay between physiological ocular maturity (slowing progression) and cumulative academic burden (lowering baseline acuity) may underlie the “low-initial slow decline” pattern in older students.

Consistent with findings from other Asian cohorts (15), our study identified maternal myopia as a significant predictor of the “low-initial slow decline” trajectory, corroborating the observation that maternal influence may be particularly important. This trend aligns with the maternal inheritance mechanism proposed by Xing et al. (16), which suggests that mitochondrial DNA variants associated with high-risk myopia are transmitted through the maternal line. This genetic evidence supports our finding that maternal visual status may play a meaningful role in predicting offspring myopia risk.

Regarding sleep habits, our results are consistent with prior research linking sleep duration to myopia progression (17). We found that students with a bedtime between 21:00 and 22:00 were more likely to maintain higher visual acuity levels compared to those sleeping after 22:00. Although the earliest bedtime group (<21:00) did not show statistical significance, this is likely attributable to limited statistical power due to a small sample size (SE = 0.503 for <21:00 vs. SE = 0.255 for 21:00–22:00). Indirectly, this scarcity of data reflects the heavy academic burden in China, where achieving a bedtime before 21:00 may be feasible for only a small proportion of students (18).

We also observed a finding that differs from some existing literature. While previous studies suggest that a low frequency of reading while lying down (i.e., better reading posture) is protective against myopia (19), our logistic regression results indicated the opposite pattern. Students who rarely read while lying down were more likely to belong to Class 3, the group with the poorest baseline visual acuity. We hypothesize that this association may reflect reverse causality rather than a direct causal effect. Specifically, students in Class 3, having already developed poor vision, may have received targeted health interventions, leading to improved reading behaviors. This interpretation is further supported by our findings on health education. In the comparison between Class 1 and Class 2, students receiving limited health education were more likely to follow the “rapid decline” trajectory (Class 1), suggesting that the absence of intervention may accelerate visual deterioration. Accordingly, the “better” reading posture observed in Class 3 may represent a consequence of post-diagnosis behavioral modification.

It is also worth discussing the non-significant factors in our model. Contrary to established consensus (20–22), variables such as gender, daily screen time, outdoor activities, and diet did not emerge as significant predictors of trajectory membership in this cohort. A plausible explanation for these null findings lies in the limitations of self-reported data. Unlike objective measurements, self-reported data collected via online surveys are prone to recall bias. Furthermore, the single-center study design implies that our sample exhibits relatively uniform socioeconomic and educational backgrounds, which may obscure the influence of these potential contributing factors.

Although refractive error was not directly measured in this study, these trajectory patterns are likely to be related to myopia development. Declines in visual acuity, particularly during critical developmental periods, may serve as early functional indicators of emerging refractive changes. Therefore, the identified trajectories may help identify children at higher risk of myopia progression, especially those in the “moderate-initial rapid decline” group. Future research incorporating direct measures of refractive error and longer-term follow-up is needed to further clarify the relationship between visual acuity trajectories and myopia progression.

### Strengths and limitations

A key strength of this study lies in its analytical approach. Rather than focusing solely on population-average trends, this study employed Latent Class Growth Modeling (LCGM) to identify subgroups of students with distinct trajectories of visual acuity development. By adopting a person-centered perspective, we were able to capture heterogeneity within the population and stratify students into clinically meaningful subgroups (23). This classification contributes to a more nuanced understanding of visual development patterns among Chinese primary school students.

Furthermore, this study integrated seven dimensions of myopia-related factors. This comprehensive framework facilitates the identification of factors associated with different trajectory classes and provides a basis for developing more targeted and stratified early intervention strategies for children at varying levels of risk.

Despite these strengths, several limitations inherent to the study design should be acknowledged. First, the use of self-reported data may introduce recall bias and potential behavioral modification due to observation (Hawthorne effect), which could affect the accuracy of the estimates. The incorporation of objective measurement tools in future studies would help address this limitation. Second, the probabilistic nature of LCGM classification implies that trajectory assignment represents an estimated classification rather than a direct observation. Moreover, although multiple covariates were adjusted for, residual confounding—such as unmeasured genetic factors or environmental conditions (e.g., ambient lighting quality)—cannot be completely ruled out. Third, the follow-up duration was limited to 1 year. While this period is sufficient to capture short-term developmental changes, it may not fully reflect long-term patterns. Therefore, longer-term longitudinal studies are needed to assess the stability of these trajectories and to determine whether individuals remain within the same trajectory group over time. Fourth, the generalizability of the findings may be limited. As the cohort was derived from four grade levels within a single primary school in Chengdu, the sample may not fully represent the broader population in terms of geographic, socioeconomic, and educational diversity. This is also reflected in the fact that, following an FDR correction, the number of significant variables decreased from five to two; we acknowledge this as a limitation and attribute it partly to the relatively homogeneous socioeconomic and behavioral background of our single-school sample, which may restrict variance in these potential predictors. Given the variability in myopia prevalence across regions and settings in China, the findings should be interpreted with caution. Finally, although this study was based on longitudinal data, causal inference remains limited. Certain behavioral factors (e.g., improved reading posture) may reflect responses to declining vision rather than antecedent causes, indicating the possibility of reverse causation. Future interventional studies, such as randomized controlled trials (RCTs), are needed to further clarify the causal mechanisms underlying myopia development.

## Conclusion

This study identified three distinct trajectories of visual acuity development among Chinese primary school students. Grade level, maternal visual status, reading habits, sleep onset time, and the frequency of health education were found to be significantly associated with these developmental patterns. These findings provide a useful reference for identifying potentially modifiable risk factors and may contribute to efforts aimed at reducing myopia risk and promoting visual health among Chinese children.

Future research is warranted to further investigate the causal relationships underlying these associations, particularly through well-designed randomized controlled trials (RCTs). In addition, extended longitudinal cohort studies are needed to validate the temporal stability of the identified trajectory classes and to explore the potential emergence of new developmental patterns over longer follow-up periods.

## Data Availability

The original contributions presented in the study are included in the article/supplementary material, further inquiries can be directed to the corresponding author.
